# Effects of Nanosecond Pulsed Electric Fields in Cell Vitality, Apoptosis, and Proliferation of TPC-1 Cells

**DOI:** 10.1155/2021/9913716

**Published:** 2021-10-13

**Authors:** Zhenguo Liu, Yawen Zou, Ying Sun, Xiaolong Chen, Xinhua Chen, Zhigang Ren

**Affiliations:** ^1^Department of Infectious Diseases, The First Affiliated Hospital of Zhengzhou University, Zhengzhou 450052, China; ^2^Gene Hospital of Henan Province, Precision Medicine Center, the First Affiliated Hospital of Zhengzhou University, Zhengzhou 450052, China; ^3^School of Medical Sciences, Zhengzhou University, Zhengzhou 450052, China; ^4^Department of Hepatobiliary and Pancreatic Surgery, The First Affiliated Hospital, School of Medicine, Zhejiang University, Hangzhou 310003, China; ^5^Key Laboratory of Pulsed Power Translational Medicine of Zhejiang Province, Hangzhou 310003, China

## Abstract

**Objective:**

To evaluate the effects of nanosecond pulsed electric fields (nsPEFs) with different pulse durations in cell vitality, apoptosis, and proliferation of TPC-1 cells, optimize pulse parameters and expand the application range of nsPEFs.

**Methods:**

The pulse duration of 0, 300 ns, 500 ns, and 900 ns is generated with nsPEF generator. CCK-8 was used to investigate the effect of nsPEFs on the viability of TPC-1 cells. Flow cytometry was used to evaluate the apoptosis of TPC-1 after pulse treatment. The effect of nsPEFs on the proliferation ability of TPC-1 cells was detected by 5-ethy-nyl-2′-deoxyuridine. The morphological changes of TPC-1 cells after pulse treatment were observed by transmission electron microscopy.

**Results:**

NsPEFs with 900 ns pulse duration can significantly affect the viability of TPC-1 cells and inhibit the proliferation ability of TPC-1 cells. In addition, nsPEFs can also induce apoptosis of TPC-1 cells.

**Conclusion:**

NsPEFs with longer pulse duration can significantly affect the biological behavior of TPC-1 cells, such as cell viability and proliferation ability, and can also induce cell apoptosis, thereby inhibiting cell growth.

## 1. Introduction

Thyroid cancer is transformed from thyroid follicular cells derived from the endoderm or thyroid C cells derived from the neural crest [1]. According to the origin and differentiation of tumors, thyroid cancer can be divided into papillary thyroid carcinoma (PTC), follicular thyroid carcinoma, medullary thyroid carcinoma, and anaplastic thyroid cancer. By far the most common form, PTC contains the classic form and 14 variants, including the high-cell and follicular variants [2], which account for approximately 85 percent of thyroid cancers [3]. Ionizing radiation is one of the risk factors for the development of PTC [4]. Over the past 20 years, the incidence of invasive PTC has increased by 9.1% per year. Due to the inherent high degree of late symptomatic disease, the sharp increase in the incidence of invasive PTC is unlikely to come from the subclinical pool [5]. Classical or high-cell variant papillary thyroid cancers with BRAF mutations show a high frequency of lymph node metastasis and recurrence after thyroidectomy and respond poorly to radiation iodine therapy [6].

When cell suspensions or tissues are exposed to a high-voltage electric field, molecules that would otherwise not easily cross the cell membrane can do so, a phenomenon known as electroporation [7]. This phenomenon exists in electrochemotherapy (ECT), irreversible electroporation (IRE), and nanosecond pulsed electric fields (nsPEFs). Compared with ECT and IRE, nsPEFs can have a profound effect on the internal structure of cells due to shorter pulse duration and higher electric field intensity [8, 9]. So far, nsPEFs have been tested in vitro and in vivo in a variety of tumors including melanoma [10, 11], squamous cell carcinoma [12], hepatocellular carcinoma [13], pancreatic cancer [14], and breast cancer [15]. Studies have shown that nsPEFs can affect organelles [16] and plasma membrane [17], increase intracellular calcium level [18], induce cell apoptosis [19], and stimulate the body to produce stress response.

In order to further expand the application scope of nsPEFs and promote the preclinical study of nsPEFs in humans, we explored the influence of nsPEFs with different pulse duration in cell vitality, apoptosis, and proliferation of TPC-1 cells.

## 2. Materials and Methods

### 2.1. Cell Culture

TPC-1, a PTC-derived cell line, was cultured in Dulbecco's Modified Eagle's Medium (DMEM, GIBCO, Carlsbad, CA, USA) containing 5% fetal bovine serum (FBS), 100 U/mL penicillin, and 100 *μ*g/mL streptomycin. The cell lines were cultured in an incubator at 37°C with 5% CO_2_.

### 2.2. nsPEF Treatment

**Figure 1:**
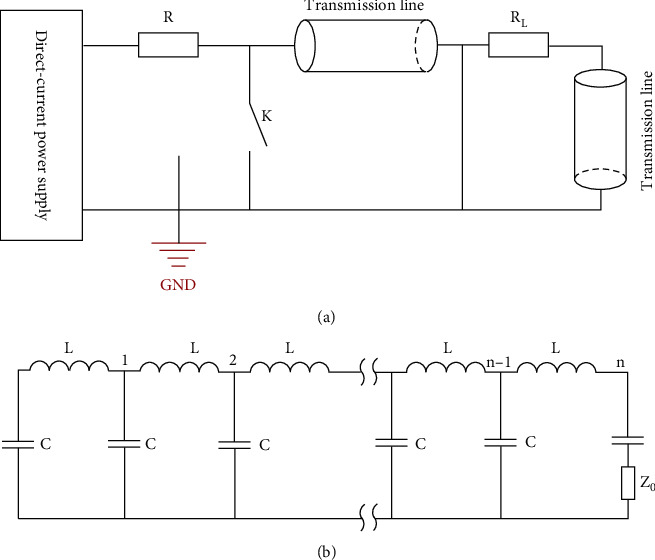
The pulse generator adopts Blumlein transmission line principle. As shown in Figure 1(a), when the closing switch *K* is not closed, the voltage of the DC supply charges the two transmission lines. After the charging is completed, the switch *K* is closed, and the energy is released to the load. When the impedance matches, the duration of the pulses loaded on the load is twice the propagation time of the electromagnetic wave in a single transmission line, and the amplitude of the pulse is the value of the charging voltage. In order to achieve adjustable pulse duration, a pulse formation network (Figure 1(b)) is adopted to simulate the two transmission lines, whose pulse duration is $2n\sqrt{LC}$, where *n* is the series of equivalent inductance (*L*) and equivalent capacitance (*C*).

We used a self-developed nsPEF generator to treat TPC-1 cells. The principle of the pulse generator is shown in Figure 1, and the shape is shown in Supplementary Figure 1A. Four cell suspensions containing 5 × 10^5^ TPC-1 cells were added to a 2 mm electroporation cuvettes (Biosmith, San Diego, California) at room temperature and then exposed to nsPEFs (field intensity 10 kV/cm, frequency 2 Hz, pulses number 600) at 0, 300 ns, 500 ns, and 900 ns, respectively (the pulse duration is specified as the interval between the rising and falling edges at 90% amplitude). Pulse waveforms and experimental apparatus for pulse processing cells are shown in Supplementary Figure 1B and 1C. After pulsed electric field treatment, the cells were inoculated in triplicate in 96-well or 24-well plates and incubated in an incubator at 37°C for different periods of time (2 to 72 h).

### 2.3. Cell Viability

We used the CCK-8 kit to evaluate the effect of nsPEFs on TPC-1 cell viability. TPC-1 cells treated with pulsed electric field were counted, and about 1 × 10^4^ cells were resuspended with 100 *μ*L DMEM containing 10% FBS and added to the 96-well plate. Each group was repeated with three duplicate wells. 10 *μ*L CCK-8 reagent (5 mg/mL, Sigma-Aldrich, St. Louis, Missouri, USA) was added to the 96-well plate at each time point after 24, 48, or 72 h of cell culture, taking care not to produce bubbles. After 2 h incubation in an incubator at 37°C, the absorbance of the sample at 450 nm was measured using a microplate meter (Type 680, Bio-Rad, Hercules, CA, USA). Finally, compared with the optical density value of the control group, the optical density value of the nsPEF treatment group was converted into the relative viability value of the cells.

### 2.4. Apoptosis Assay

The apoptosis of TPC-1 cells after pulsed electric field treatment was assessed using FITC-Annexin V Apoptosis Detection Kit I (BD, Oxford, UK). Cells positive for FITC-Annexin V and negative for propidium iodide (PI) staining experienced early apoptosis. Late apoptosis occurred in cells that were both positive for FITC Annexin V and PI staining. Cells with negative Annexin V and PI staining of FITC were normal cells. TPC-1 cells treated with pulsed electric field were left standing for 1 h, then resuspended and centrifuged and mixed with Annexin-FITC binding solution and propidium iodide (PI) staining solution. Finally, after 20 minutes of dark incubation, the cells were analyzed by flow cytometry (BD, Oxford, UK).

### 2.5. EdU Proliferation Assay

The proliferative ability of pulsed TPC-1 cells was examined using the 5-ethy-nyl-2′-deoxyuridine (EdU) assay kit (Ribobio). As a thymine nucleoside analogue, EdU is able to replace thymine during cell proliferation by infiltrating into replicating DNA molecules. The cell proliferation rate can then be measured by double labeling the nucleus in combination with nuclear markers (e.g., Hoechest 33342). TPC-1 cells treated with pulsed electric field were cultured in medium-containing EDU (final concentration of 10 *μ*m) at 37°C for 2 h, and then the cells were washed with PBS 1 ~ 2 times, each time for 5 min. After adding 1 mL 4% paraformaldehyde, the cells were fixed at room temperature for 15 min. After removing the fixed solution, the samples were washed with PBS and then incubated with PBS containing 0.5% Triton X-100 for 10 minutes. Hoechst 33342 was stained for 30 min. After washing, the staining results were observed by inverted fluorescence microscope (Nikon Inverted Research Microscope ECLIPSE Ti). For each staining result, five random fields were imaged at ×20 magnification. The image was analyzed with Image-Pro Plus software. EdU incorporation rate was expressed as the ratio of the number of EdU-positive cells to the total number of cells in each field.

### 2.6. Transmission Electron Microscope (TEM) Analysis

The morphological changes of TPC-1 cells treated with pulsed electric field were observed by transmission electron microscopy. TPC-1 cells treated with pulsed electric field were fixed with 2.5% glutaraldehyde for 2 h and then washed with PBS. Cell samples were immersed in PBS containing 1% OSO4 for 3 hours and then washed with PBS. The samples were dehydrated with a series of different concentrations of ethanol (30%, 50%, 70%, 80%, 90% and 95%) and washed three times with 100% acetone. After the sample is embedded, ultra-thin slices (70 nm) were obtained using an ultra-thin slicer (LEICA EM UC7). After staining the samples with uranium acetate (5%) and lead citrate (1%), the samples were observed by TEM (HitachiH-7650).

### 2.7. Statistical Methods

Statistical analysis was performed using Windows SPSS 17.0 (SPSS, USA). All data are expressed as mean ± standard deviation. The differences between the two groups were measured using the *t*-test of the two samples. One-way analysis of variance was used for comparisons between groups. If *p* is less than 0.05, the result is statistically significant.

## 3. Results

### 3.1. NsPEFs with 900 ns Pulse Duration Decreased the Viability of TPC-1 Cells

**Figure 2:**
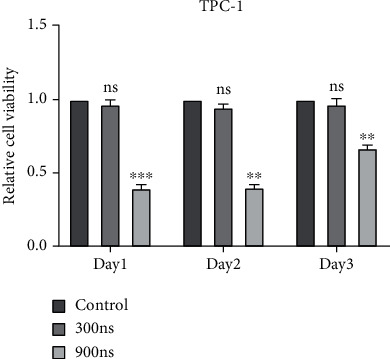
NsPEFs decreased the cell viability of TPC-1 cells. TPC-1 cells were treated with pulsed electric fields of 0 ns, 300 ns, and 900 ns pulse duration. Then, CCK-8 was used to analyze the effect of nsPEFs on the viability of TPC-1 cells. ^∗^*p* < 0.05; ^∗∗^*p* < 0.01; ^∗∗∗^*p* < 0.001.

By CCK-8, we detected the viability of TPC-1 cells at 24 h, 48 h, and 72 h after nsPEF treatment. As shown in Figure 2, compared with the control group, nsPEFs with a pulse duration of 300 ns did not affect the viability of TPC-1 cells, but nsPEFs with a pulse duration of 900 ns significantly affected the viability of TPC-1 cells. In addition, cell viability increased slightly 72 h after pulsed electric field treatment, but it was still lower than the normal control group.

### 3.2. NsPEFs with 900 ns Pulse Duration Induced Late Apoptosis/Necrosis of TPC-1 Cells

**Figure 3:**
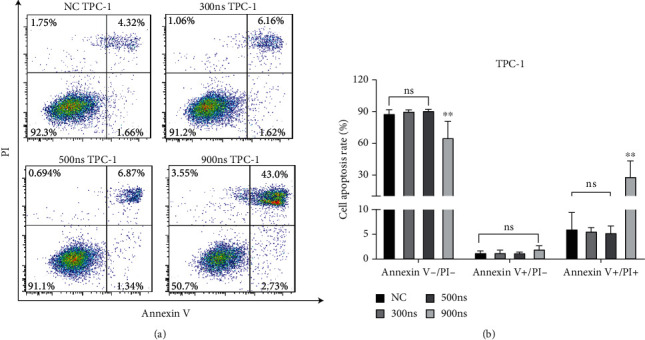
NsPEFs affected the apoptosis of TPC-1 cells. (a) After treated with different parameters of nsPEFs, TPC-1 cell apoptosis was detected by flow cytometry. (b) Analysis showed that nsPEFs with 900 ns pulse duration induced late apoptosis/necrosis. ^∗^*p* < 0.05; ^∗∗^*p* < 0.01; ^∗∗∗^*p* < 0.001.

In order to evaluate whether nsPEFs has an apoptotic effect on TPC-1, we used flow cytometry to detect the apoptosis/necrosis rates of cells treated with different parameters of the pulsed electric field. As shown in Figure 3, compared with the control group, nsPEFs with a pulse duration of 900 ns induced late apoptosis/necrosis of TPC-1 cells, but had no effect on early apoptosis rate. In addition, nsPEFs with pulse duration of 300 ns or 500 ns did not change the apoptotic/necrosis rate of TPC-1 cells.

### 3.3. NsPEFs with 900 ns Pulse Duration Inhibited the Proliferation of TPC-1 Cells

**Figure 4:**
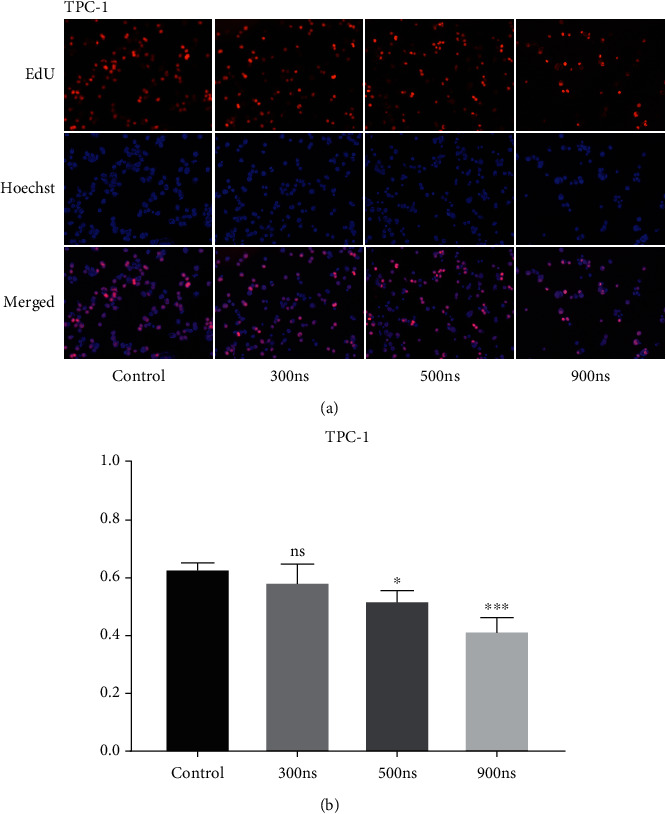
NsPEFs inhibited the proliferation of TPC-1 cells. (a) After nsPEFs treatment with different parameters and staining, the cells were observed under an inverted microscope. (b) NsPEFs with longer pulse duration significantly inhibited cell proliferation. ^∗^*p* < 0.05; ^∗∗^*p* < 0.01; ^∗∗∗^*p* < 0.001.

The proliferation ability of TPC-1 cells under different pulses was detected by EDU. As shown in Figure 4, compared with the control group, nsPEFs with pulse duration of 300 ns did not affect cell proliferation. The proliferation of TPC-1 cells was significantly inhibited by nsPEFs with pulse duration of 500 ns or 900 ns. In addition, nsPEFs with longer pulse durations can achieve higher proliferation inhibition.

### 3.4. NsPEFs with 900 ns Pulse Duration Induced Apoptosis in TPC-1 Cells

**Figure 5:**
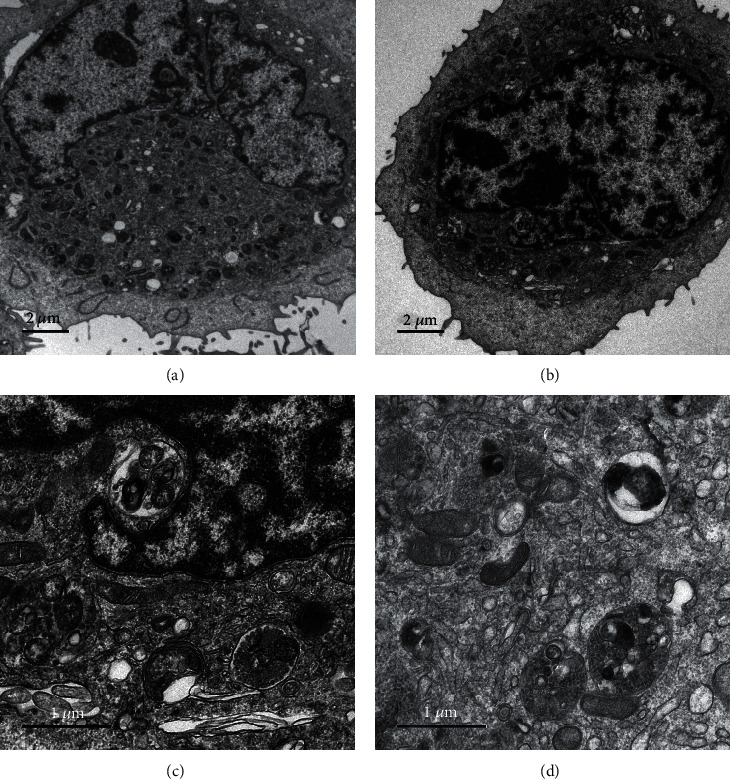
TPC-1 electron microscope image after nsPEF processing. For (a) and (b), the magnification is 2500, while for (c) and (d), the magnification is 10000.

The cell morphology after pulsed electric field treatment was clearly observed by TEM. As shown in Figure 5, the chromatin is concentrated into clumps and bordered to the nuclear membrane in Figures 5(a) and 5(b). The nuclear membrane appears to roll inwardly and shrink. The microvilli are shed and reduced. In Figures 5(c) and 5(d), the cytoplasm is concentrated, mitochondria are enlarged with increased cristae, and there are many small vacuoles in the cytoplasm. This proves that nsPEFs can induce apoptosis of TPC-1 cells.

## 4. Discussion

NsPEFs have attracted increasing attention from researchers involved in cancer therapy because of their ability to fight against tumor cells in a novel way that induces cell death. In this study, we treated TPC-1 cells with nsPEFs of different pulse durations, further confirming that nsPEFs with long pulse durations can reduce the viability and proliferation of TPC-1 cells. Flow cytometry and transmission electron microscopy confirmed the occurrence of apoptosis. This study shows that nsPEFs with long pulse duration can be effective in the treatment of thyroid cancer, which provides a new approach for the treatment of thyroid cancer.

The ablation effect of nsPEFs depends on the field intensity, pulse duration, and pulse number. In pancreatic cancer, liver cancer, breast cancer cells, and animal experiments, nsPEFs have shown a dose-dependent effect on tumor cells in terms of field intensity and pulse number. The occurrence of plasma membrane effect and intracellular effect depends on the duration of pulse [20]. Our study shows that nsPEFs with 900 ns pulse duration can significantly affect the viability and proliferation of TPC-1 cells. Ren et al. found in the in vitro experiment of pancreatic cancer that nsPEFs could inhibit the expression of cyclin by inhibiting the NF-*κ*B signaling pathway, thereby inhibiting cell proliferation [21]. In addition, in vivo animal experiments, Chen et al. [22] found that the inhibition effect of multiple low-dose pulsed electric fields was better than that of a single high-dose pulsed electric field, which may be attributed to the effect of macrophages. Encouragingly, Yin et al. [13] also found that nsPEFs could also reduce extrapulmonary metastasis of liver cancer.

Cell apoptosis has always been the focus of research on nsPEF ablation for tumors. Externalization of phosphatidylserine is also a marker of apoptosis [23]. Our flow cytometry results indicated that significant phosphatidylserine ectropion could be observed on nsPEFs with longer pulse duration. At the same time, the results of cell electron microscopy showed contraction of nuclear membrane, chromatin agglutination, and vacuoles in the cytoplasm, which confirmed the occurrence of cell apoptosis. In vivo ablation of rat liver cancer models, Nuccitelli et al. [16] observed rapid swelling of mitochondria, endoplasmic reticulum, and Golgi apparatus. The formation of irregular holes in the plasma membrane is caused by the pulsed electric field acting on charged molecules in the plasma membrane [24]. In addition, the decrease of mitochondrial membrane potential and the release of cytochrome may play an important role in cell apoptosis [19, 20]. Mitochondria-dependent pathways may play an important role in nsPEF treatment of human HepG2 cells [25].

In vivo animal studies have shown that nsPEFs have many advantages. First, it significantly reduced muscle contractions during ablation compared to IRE [26]. Second, it preserves the integrity of the supporting tissue [27]. In the end, tissue after nsPEFs ablation repaired and healed more quickly. In the ablation experiment of the rat liver cancer model, Chen et al. [28] observed the proliferation of the normal liver tissue by contrast enhanced ultrasound, which undoubtedly indicates that nsPEFs are a relatively mild method for body ablation and are different from the direct killing effect of thermal ablation. In addition, the infiltration of immune cells and the presence of granzyme B expressing cells within a few days after ablation indicated the occurrence of an adaptive antitumor immune response [28]. However, no antitumor immunity was found in an animal study of melanoma [11]. Therefore, whether the immune response is produced or not needs further research.

More recently, combining nsPEFs with other treatments has also attracted interest. For example, drugs such as doxorubicin [29], paclitaxel [30], everolimus [31], and PD-L1 blockers [32] are combined with nsPEFs, and even infrared radiation is combined with nsPEFs.

## 5. Conclusions

In this study, we investigated the effect of nsPEFs with different pulse durations on TPC-1 cells. NsPEFs with 900 ns pulse duration can significantly affect the viability and proliferation ability of TPC-1 cells and induce their apoptosis, thus inhibiting the growth of TPC-1 cells. This study contributed to the adjustment of pulse parameters and expanded the ablation target of nsPEFs.

## Data Availability

The [data type] data used to support the findings of this study are available from the corresponding author upon request.

## Supplementary Materials

Supplementary Materials Supplemental Figure 1: (a) the nanosecond pulsed electric field (nsPEF) generator. (b) The waveform of nsPEF (field intensity 10 kV/cm, pulse duration 300 ns). (c) The ablation equipment for cell experiment.
